# Mechanisms of growth inhibition of *Phytomonas serpens* by the alkaloids tomatine and tomatidine

**DOI:** 10.1590/0074-02760140097

**Published:** 2015-02

**Authors:** Jorge Mansur Medina, Juliany Cola Fernandes Rodrigues, Otacilio C Moreira, Geórgia Atella, Wanderley de Souza, Hector Barrabin

**Affiliations:** 1 Instituto de Bioquímica Médica Leopoldo de Meis; 2 Laboratório de Ultraestrutura Celular Hertha Meyer, Instituto de Biofísica Carlos Chagas Filho, Universidade Federal do Rio de Janeiro, Rio de Janeiro, RJ, Brasil; 3 Instituto Nacional de Ciência e Tecnologia de Biologia Estrutural e Bioimagem, Rio de Janeiro, RJ, Brasil; 4 Instituto Nacional de Metrologia, Qualidade e Tecnologia, Rio de Janeiro, RJ, Brasil; 5 Núcleo Multidisciplinar de Pesquisa em Biologia, Universidade Federal do Rio de Janeiro, Duque de Caxias, RJ, Brasil; 6 Laboratório de Biologia Molecular e Doenças Endêmicas, Instituto Oswaldo Cruz-Fiocruz, Rio de Janeiro, RJ, Brasil

**Keywords:** Phytomonas, tomatine, tomatidine, lipids, trypanosomatids

## Abstract

Phytomonas serpens are flagellates in the family Trypanosomatidae that parasitise the tomato plant (Solanum lycopersicum L.), which results in fruits with low commercial value. The tomato glycoalkaloid tomatine and its aglycone tomatidine inhibit the growth of P. serpens in axenic cultures. Tomatine, like many other saponins, induces permeabilisation of the cell membrane and a loss of cell content, including the cytosolic enzyme pyruvate kinase. In contrast, tomatidine does not cause permeabilisation of membranes, but instead provokes morphological changes, including vacuolisation. Phytomonas treated with tomatidine show an increased accumulation of labelled neutral lipids (BODYPY-palmitic), a notable decrease in the amount of C_24_-alkylated sterols and an increase in zymosterol content. These results are consistent with the inhibition of 24-sterol methyltransferase (SMT), which is an important enzyme that is responsible for the methylation of sterols at the 24 position. We propose that the main target of tomatidine is the sterols biosynthetic pathway, specifically, inhibition of the 24-SMT. Altogether, the results obtained in the present paper suggest a more general effect of alkaloids in trypanosomatids, which opens potential therapeutic possibilities for the treatment of the diseases caused by these pathogens.

Trypanosomatid parasites are protozoa that are agents of human diseases, such as Chagas disease, African trypanosomiasis and leishmaniasis. Many plants also harbour trypanosomatids, which primarily reside in the xylem, phloem tubes (França 1914, Holmes 1925, Harvey & Lee 1943, Dollet 2001 ), fruits and/or seeds (Jankevicius et al. 1987, 1989, 1993 , Conchon et al. 1989, Sánchez-Moreno et al. 1995) of infected plants. The presence of the pathogens in the fruits or seeds may yield local pathological symptoms, but never systemic symptoms. Trypanosomatids are agents of plant diseases, such as Hartrot of the coconut (Parthasarathy et al*.* 1976), Marchitez of oil palm (Dollet et al. 1977), wilt of the ornamental ginger *Alpinia purpurata *(Gargani et al. 1992) and coffee tree phloem necrosis. The commercial importance of these crops emphasises the need for more research on these devastating diseases. Such diseases are likely transmitted through the bite of phytophagous insects (Camargo et al. 1990 , Camargo & Wallace 1994, Redman et al*.* 1995). Tomatoes can be infected by *Phytomonas serpens* without apparent pathological effect, but the fruits present yellow spots that are consequently of low commercial value.

Plants produce compounds that provide chemical defences against pests, pathogens and invasion by neighbouring plants. These compounds include sterols and triterpenes that are formed by cyclisation of 2,3-oxidosqualene. Sterols and triterpenes accumulate as glycoside conjugates and include glycoalkaloids, which are commonly referred to as saponins. Saponins possess antimicrobial and antifungal activities that act as plant defences. Saponins act by increasing membrane permeability because of their ability to form complexes with cholesterol. Tomato plants (*Solanum lycopersicum *L.) produce tomatine, which is a tetrasaccharide linked to the 3-OH group of the aglycone tomatidine (Fig. 1). Immature green tomatoes contain up to 500 mg of tomatine/kg of fresh fruit weight (Friedman 2002 ). The compound is largely degraded as the tomato ripens until it reaches levels in mature red tomatoes of 5 mg/kg of fresh fruit weight. Glycoalkaloids from potatoes (α-chaconine, α-solanine), eggplant (α-solamargine, solasonine) and tomatoes (tomatine) are growth inhibitors of *Trypanosoma cruzi*, strain EP, in liver infusion tryptose medium (Chataing et al. 1998).

**Fig. 1:**
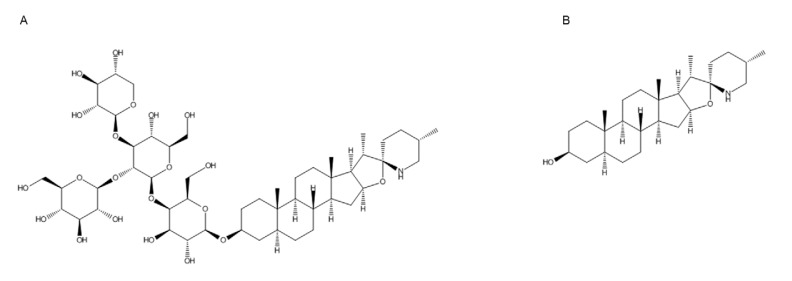
structure of tomatine (A) and tomatidine (B).

The major sterols of the trypanosomatids are C_28_-ergostane (24-methyl group) and C_29_-stigmastane (24-ethyl) types, in which an extra methyl or ethyl group is added to carbon-24 of the sterol side chain. In contrast, animals synthesise C_27_ cholestane-based members of the steroid family. The alkylations of the sterol side chain are performed by two distinct 24-sterol methyltransferases (SMT), SMT type 1 (SMT1) and type 2 (SMT2). Various azasterols with a nitrogen substitution in the side chain inhibit C-24 transmethylation reactions involving *S*-adenosyl methionine as the methyl donor and a ∆^24(25)^-sterol or ∆^24(24`)^-sterol substrate. 24-SMT is present in fungi, plants and trypanosomatids, but it is absent in mammals, which makes this enzyme an attractive target for chemotherapeutic agents against pathogens (Haughan et al. 1995, Rodrigues et al. 2002, Roberts et al. 2003, de Souza & Rodrigues 2009).

Here, we investigated the toxicity of tomatine and its aglycone, tomatidine, against *P. serpens*. Both compounds inhibit the growth of *Phytomonas*, but they utilise different mechanisms. The glycoalkaloid tomatine disrupts the plasma membrane, which kills the parasite and tomatidine induces notable morphological changes in the parasite, apparently due to the inhibition of alkylated sterols synthesis.

## MATERIALS AND METHODS

*Materials* - Tomatine and tomatidine were acquired from Sigma Chemical Co Stock solutions of both alkaloids were prepared in dimethyl sulfoxide (DMSO). DMSO alone had no effect on cell proliferation. BODIPY FL C16 was obtained from Molecular Probes (USA).

*Cell cultures *- *P. serpens* (CT.IOC 189) were grown at 27ºC in a medium containing 20 g/L sucrose, 20 g/L KCl, 3 g/L peptone and 1 mg/L folic acid, supplemented with 10 mg/L haemin and 10% (v/v) foetal bovine serum.

*Effects on growth* - Cultures were initiated at a cell density of 1 × 10^6^ cells/mL and drug was added 24 h later, when the number of parasites in the cultures reached approximately 5 × 10^6^ cells/mL. Cell densities were evaluated daily in a Neubauer chamber after 96 h of growth. The half maximum inhibitory concentration (IC_50_) values were calculated using GraphPad 5.0 software.

*Cell permeabilisation assay* - Promastigotes of *P. serpens* were harvested after two days of growth, washed in phosphate buffered saline (PBS), pH 7.2 and counted in a Neubauer chamber. Cells were incubated with tomatine, tomatidine or digitonin at the concentrations indicated in the Figure legends for 10 min. Cell suspensions were centrifuged at 12,000 rpm in an Eppendorf centrifuge for 5 min. The amount of pyruvate kinase (PK) (cytosolic enzyme) in solution was measured in 0.5-mL aliquots of supernatant using a coupled assay in media containing 50 mM Tris HCl pH 8.0, 5 mM MgCl_2_, 1 mM ADP, 1 mM phosphoenolpyruvate, 100 mM KCl, 0.25 mM β-NADH and 0.1 U lactate dehydrogenase in final volume of 2 mL. Changes in absorbance at 340 nm were monitored at 2-min intervals to estimate the rate of β-NADH consumption.

*BODIPY-palmitic acid labelling* - Cells were grown for 24 h and 5 µM BODIPY FL C16 was added to 2-mL aliquots of culture, in which 50 µM tomatidine was or was not included. After 48 h, the top 1.5 mL portion of the culture was withdrawn and washed twice with PBS. Cells were fixed using 4% formaldehyde and observed under a fluorescence microscope (Axiovision; Carl Zeiss) using a 505 nm excitation filter and a 515 nm emission filter. The images were further processed using Adobe Photoshop CS2 (Adobe Systems Inc).

*Transmission electron microscopy (TEM)* - Growing parasites were treated with tomatidine after 24 h and cells were harvested 48 h later, washed with PBS and fixed using 2.5% glutaraldehyde grade I in 0.1 M cacodylate buffer for 2 h at room temperature (RT). Cells were washed twice with 0.1 M cacodylate buffer, pH 7.2 and post-fixed in the dark using 1% osmium tetroxide, 0.8% potassium ferrocyanide and 5 mM CaCl_2_ in cacodylate buffer for 30 min at RT. Cells were washed with cacodylate buffer, dehydrated in acetone solutions of increasing concentrations and embedded in epoxy resin (Polybeb 812). Ultrathin slices were obtained using a Reichert ultramicrotome, collected on 300-mesh cupper grades and contrasted with 5% uranile for 45 min and lead citrate for 5 min, according to the protocol of Reinolds. The samples were analysed using an electromicroscope Zeiss 900 and Jeol 1200, operating at 80 KV.

*Sterol analyses* - Parasite lipids were extracted using the method of Bligh and Dyer (1959). Briefly, *Phytomonas* were washed with PBS and 5 x 10^7 ^cells were intermittently agitated for 1 h with ~4 mL of a methanol:chloroform:water (2:1:0.8) mixture. The tubes were centrifuged at 2,000 *g* (15 min) and the supernatant, which contained the lipids, was separated. The pellets were extracted one more time and the supernatants were combined. A two-phase separation was forced by the addition of 1 mL water and 1 mL chloroform. Samples were agitated and the organic (bottom) phase was separated and dried under a dry N_2_ stream.

The residue was saponified using alcoholic KOH, extracted and dissolved in pure chloroform. Samples were treated with trimethylchlorosilane and analysed using gas chromatography with detection by mass spectrometry (GC-MS). A GC-MS Shimadzu gas chromatograph was used, model GP2010 Plus, with an RTX^(r)^-5 MS (5% phenyl 95% dimethylpolysiloxane) column from Restek^(r)^ (30 m x 0.25 mm x 0.25 µm). The injector was maintained at 250ºC with a 1:1 split flow ratio. The column oven temperature was maintained at 170ºC for 5 min, then increased to 250ºC with a heating rate of 20ºC min^-1^ and finally to 280ºC, with a high rate of heating of 5ºC min^-1^, which was maintained for 20 min. Helium was used as a carrier gas, with a linear velocity of 41.9 cm s^-1^. Volumes of 1 µL were injected into the chromatograph for analysis.

The MSdetector was equipped with an electron (EI-70 eV) ionisation source and a quadrupole mass analyser, operated in SCAN mode (50-700 µm.a.). The interface was maintained at 230ºC and the ion source was maintained at 200ºC. The components were identified by comparing their mass spectra with spectra in the NIST05 library stored in the computer controlling the mass spectrometer and a critical analysis of fragmentation patterns. Retention indices were also used to confirm the identity of the peaks in chromatograms.

*Statistical analysis *- Data are expressed as means ± standard error from at least three independent experiments. Statistical significance was calculated using two-way ANOVA followed by Bonferroni's post-test. A difference was considered to be statistically significant when p ≤ 0.05. Analyses were performed using GraphPad 5.0 software.

## RESULTS

**Fig. 2A, B:**
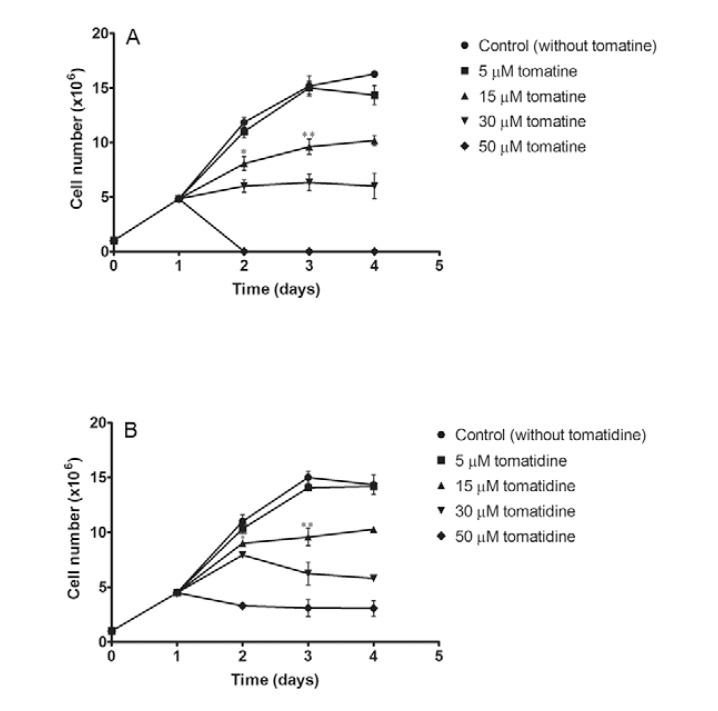
growth curve of Phytomonas serpens in the presence of tomatine and tomatidine. Promastigotes (1 × 106/mL) were grown in medium supplemented with 10% foetal bovine serum at 27ºC. After 24 h of growth, tomatine or tomatidine were added at the indicated concentrations. Each point represents the mean ± standard error of the means of three independent experiments. *: p < 0.05; **: p < 0.001 (2-way ANOVA with Bonferroni's post test). The half maximum inhibitory concentration values obtained after 48 h of treatment were 9.9 μM (for tomatine) and 14.2 μM (for tomatidine). Dimethyl sulfoxide alone had no effect on cell proliferation (data not shown).

*Effects of tomatine and tomatidine on the growth rate of* *Phytomonas* - Tomatine induced a dose-dependent inhibition of *Phytomonas* growth with an IC_50_ of 9.9 µM after 48 h of incubation. Fig. 2A shows that the presence of 50 µM tomatine in the culture media caused the death of all cells. Tomatidine also produced a dose-dependent inhibition of growth (IC_50_ = 14.2 µM). In contrast, 50 µM tomatidine stopped the growth of *Phytomonas* in culture (Fig. 2B), apparently without killing the cells. This behaviour could be a result of the inhibition of the cell cycle *via* blockade of nucleus or kinetoplast separation during the mitotic process. However, the results obtained by the labelling of DNA with 4'-6-diamidino-2-phenylindole excluded this hypothesis because no increase in the number of cells containing more than one nucleus or kinetoplast was observed (not shown). Additionally, the fluorescence intensity of these organelles was comparable in normal and treated parasites, which suggests that the amount of DNA per cell was not changed.

**Fig. 3:**
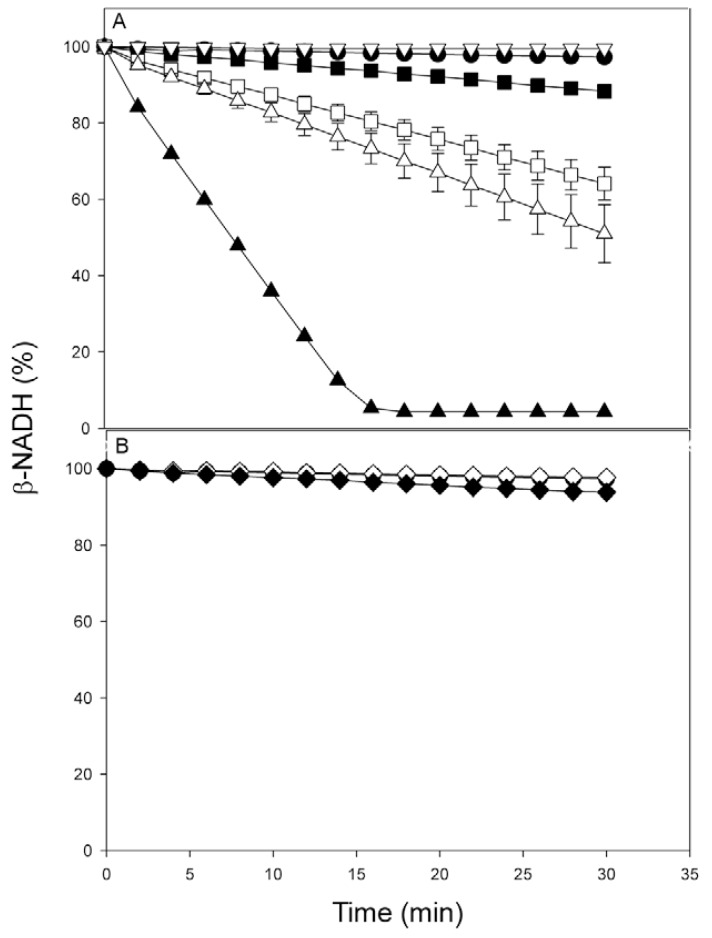
tomatine induced pyruvate kinase (PK) leakage from Phytomonas serpens. Cells were harvested after two days of growth and resuspended in phosphate buffered saline at the concentrations indicated below. The tomatine (A) or tomatidine (B) was added and after 10 min, the cells were spun down and PK was measured in 0.5 mL of the supernatant (see Materials and Methods). : control without alkaloids; : 1 x 106 cell/mL treated with 10 μM tomatine; □: 3 x 106 cell/mL treated with 10 μM tomatine; : 3 x 106 cell/mL treated with 200 μM digitonin; ▲: 30 mU of purified PK; : 3 x 106 cell/mL treated with 10 μM tomatine and measured in the absence of lactate dehydrogenase; º : 1 x 106 cell/mL treated with 10 μM tomatidine; : 3 x 106 cell/mL treated with 100 μM tomatidine. Each set of curves was repeated three times and the Figure is representative of these experiments.

*Permeabilisation of the cell membrane* - Some glycoalkaloids interact with sterols, such as cholesterol and ergosterol, to disrupt the membrane and cause a loss of cellular content (Morrissey & Osbourn 1999). The leaking of PK (which is a cytosolic enzyme) after treatment of *Phytomonas* with either tomatine or tomatidine was measured to test this hypothesis. Tomatine (10 µM) was very effective in permeabilising the cellular membrane (Fig. 3A). The amount of PK released was proportional to the amount of cells and the release was very similar to the extent of release induced by 200 µM digitonin. This result supports that tomatine kills cells *via* permeabilisation, which is similar to other glycoalkaloids.

In contrast, tomatidine concentrations as high as 100 µM did not trigger any membrane leakage (Fig. 3B), which implies a different mechanism of cell poisoning.

**Fig. 4:**
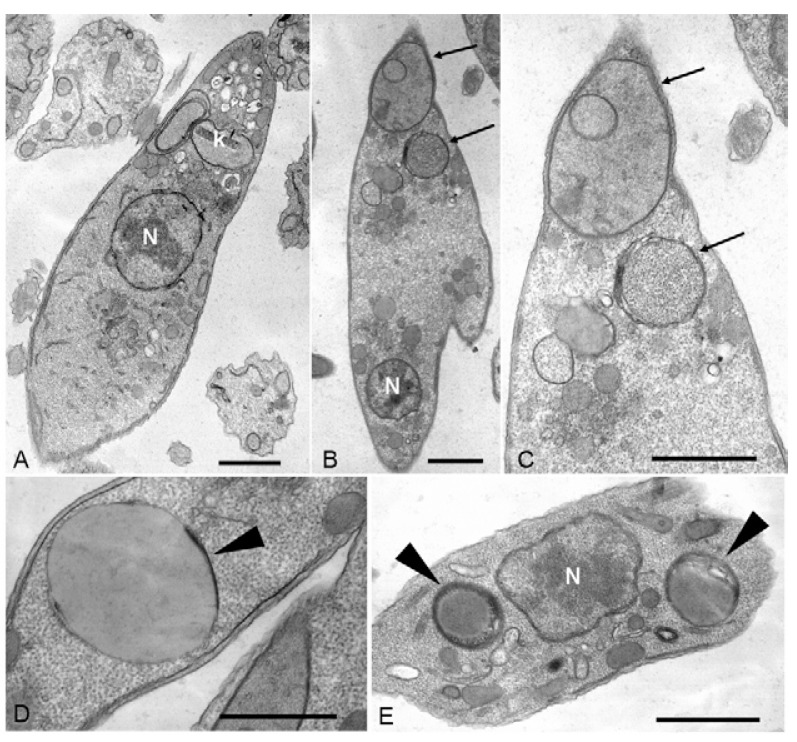
ultrastructural alterations of Phytomonas serpens induced by tomatidine. A: ultrathin section of P. serpens without treatment which presents a normal ultrastructure of organelles such as kinetoplast (k) and nucleus (N); B-D: electron micrograph of P. serpens treated with 30 μM tomatidine for 48 h presenting many vacuoles (arrows), in some cases localised to the posterior region of the parasite; E: after treatment with 50 μM tomatidine for 48 h, it is possible to observe the presence lipid bodies (arrowheads). Bars = 1 μM (A-C, E) and 0.25 μM (D).

*Cell morphology alterations* - Optical microscopy of *Phytomonas* treated with tomatidine and stained using Giemsa revealed several clear spots, like "holes", along the cell body (not shown). Treated cells observed under TEM revealed the presence of large rounded bodies, some with multiple membrane layers (Fig. 4B-D) and others with the appearance of lipid bodies (Fig. 4D-E, arrow heads). No significant changes in nuclear or kinetoplast morphologies were observed.

**Fig. 5:**
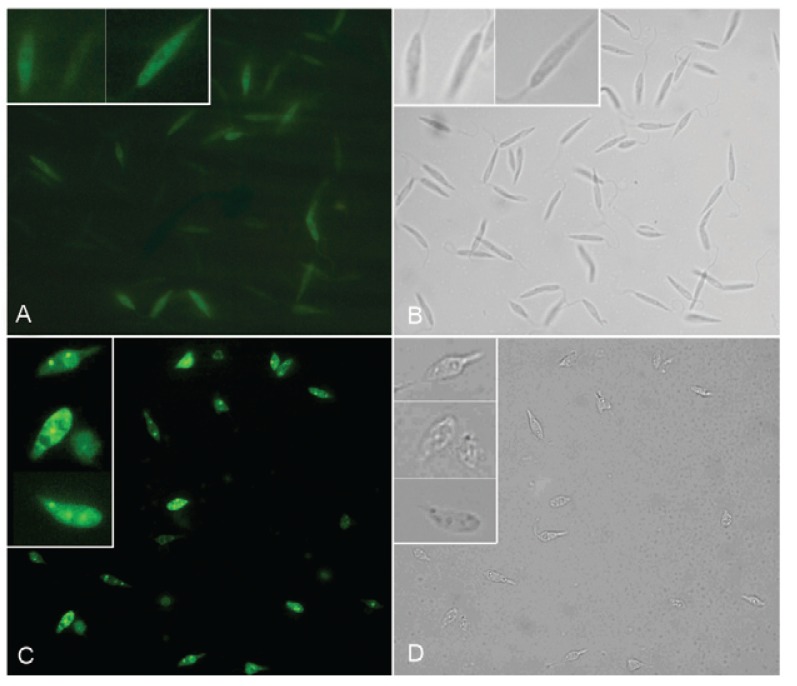
fatty acid incorporation in Phytomonas serpens vacuoles. P. serpens was grown for two days in the presence of 5 μM labelled BODIPY-palmitic acid (green) and in the absence (A) or presence (B) of 50 μM tomatidine as described in Materials and Methods, then harvested and resuspended in phosphate buffered saline for fluorescence microscopy. Right panels are images obtained by interferential contrast microscopy. The inserts represents typical cells in detail. Bars = 10 μM; inserts = 0.25 μM.

*Fatty acid incorporation in Phytomonas* - The microscopy data suggested the formation of vacuoles that contained a clear material with the appearance of lipids. Assays of fatty acids incorporation using the fluorescent analogue BODIPY were performed to confirm this hypothesis. Tomatidine-exposed cells presented a build-up of label lipids in rounded structures in the cytoplasm (Fig. 5). These spots had a similar size and distribution to the vacuoles observed in Fig. 4, which indicated that tomatidine may perturb lipid metabolism and membrane organisation.

*Analysis of lipid composition* - Lipids extracted from *Phytomonas* were analysed using GC-MS (Table). The most abundant *Phytomonas* sterols were cholesterol (14.8 %), ∆^7^- ergosterol (15 %), ∆^7^-sitosterol (37.9 %) and 24 ethyllophenol (20 %). *Phytomonas* and other trypanosomatids do not have a complete pathway for cholesterol synthesis. Therefore, the presence of cholesterol must be a consequence of its uptake from the culture media.

**TABLE:** Effectsof tomatidine (Td) on the free sterol contents of Phytomonas serpens

Sterol	Molecular structure	Retention time (min)	Control	(5 µM)	Tomatidine (15 µM)	(50 µM)
cholest-5-en-3β-ol (exogenous cholesterol)	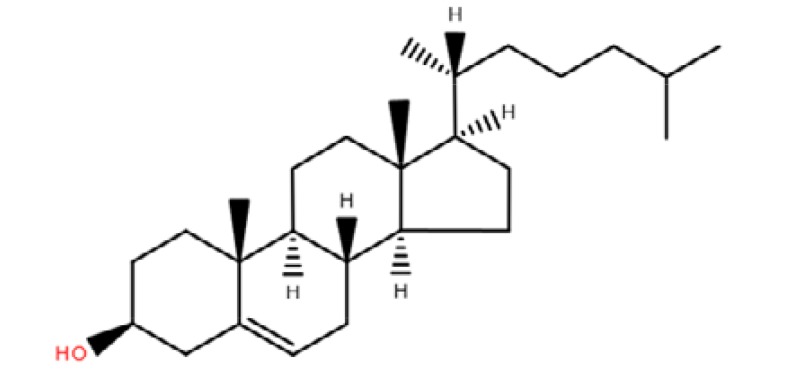	19.31	14.8	28	48	48.6
cholesta-8,24-dien-3β-ol (zymosterol)	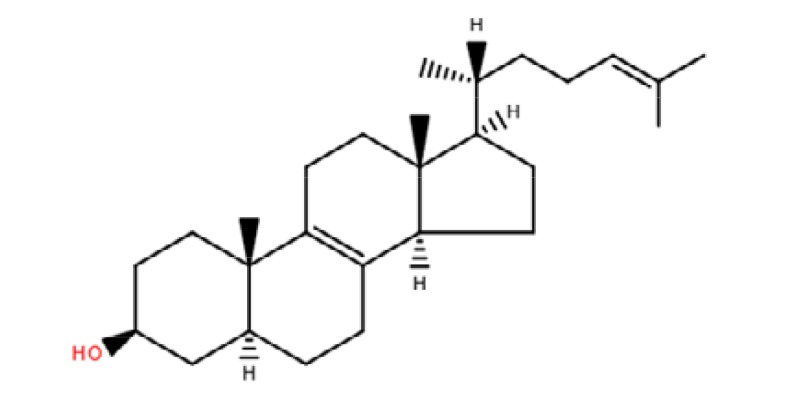	20.44	ND	2.1	17.8	17.3
cholesta-7,24-dien-3β-ol	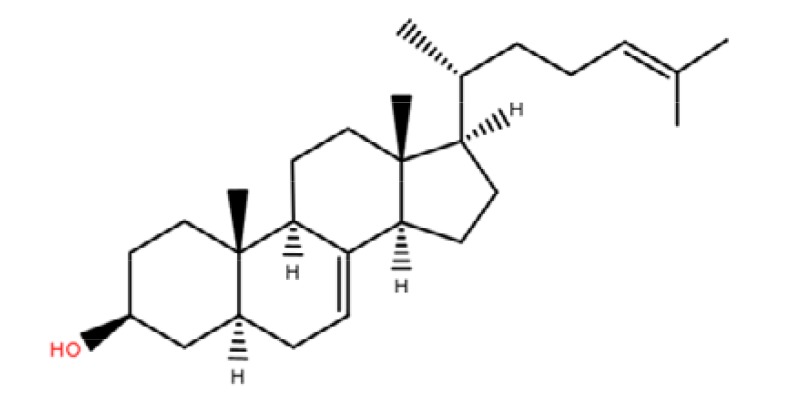	21.22	ND	54	22.9	4.1
4-methylcholesta-8,24- dien-3β-ol (4α-methyl-zymosterol)	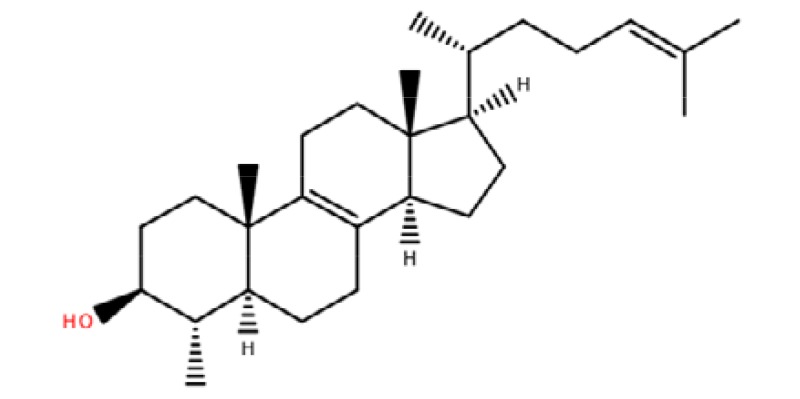	22.28	ND	ND	ND	12.7
ergost-7-en-3β-ol (∆^7^-ergosterol)	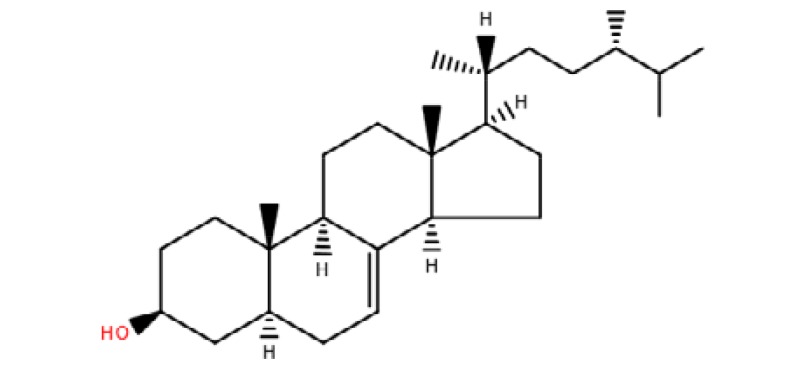	22.63	15	2.7	5.3	6.3
Not identified		23.14	ND	9.1	ND	ND
stigmast-7-en-3β-ol (∆^7^-sitosterol)	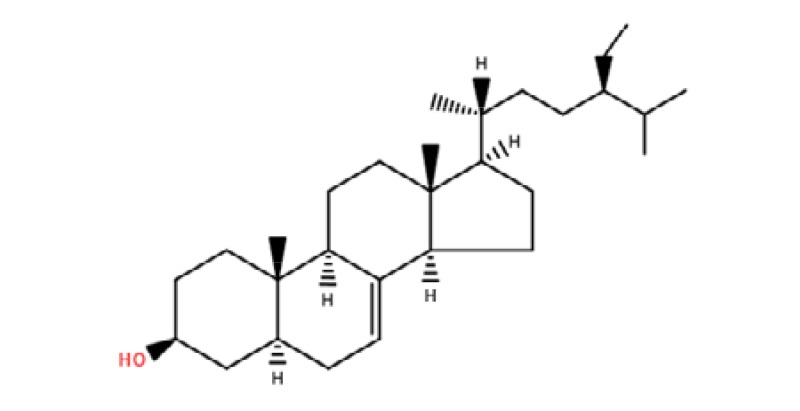	24.88	37.9	4.1	6	11
∆^7^-avenasterol	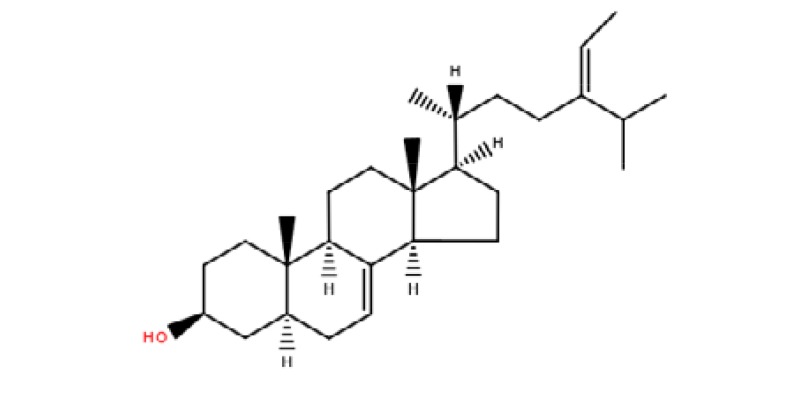	25.38	5.7	ND	ND	ND
4α-methyl-5a-stigmast- 7-en-3 β-ol (24 ethyllophenol)	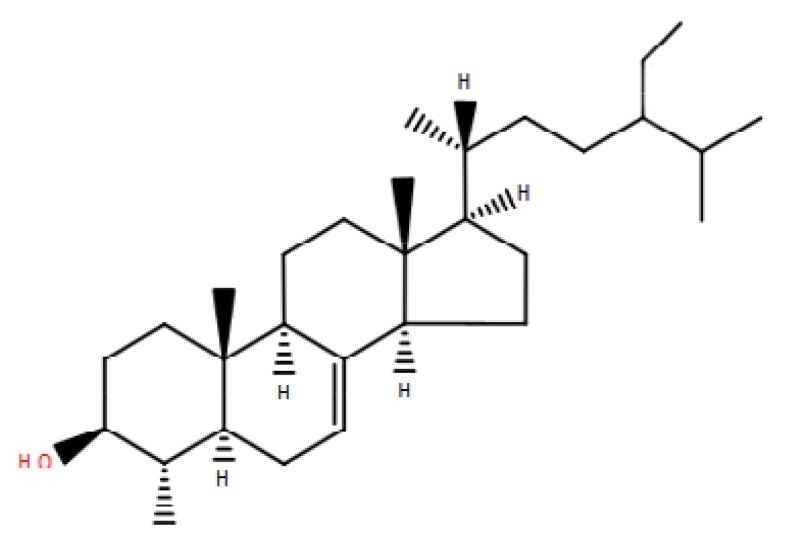	27.62	20	ND	ND	ND
4α-methyl-5 α -stigmasta- 7,24(24^1^)-dien-3β-ol (24 ethylidenelophenol)	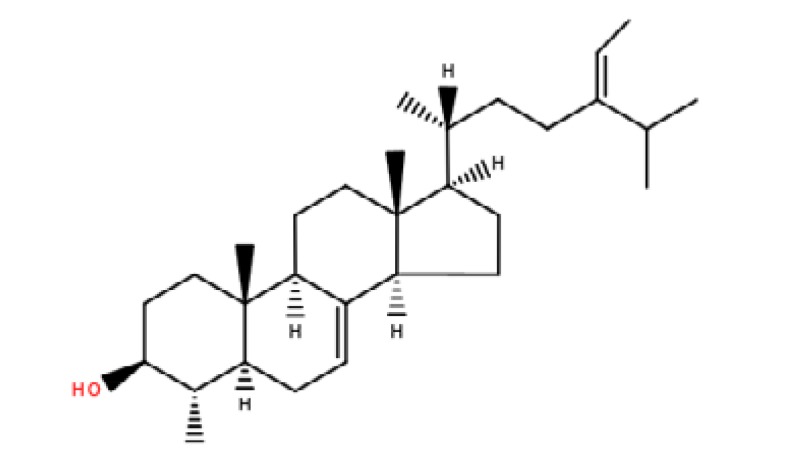	28.29	6.6	ND	ND	ND

total lipids were extracted from the control cells and cells treated with tomatidine for 48 h. The extracted lipids were saponified and analysed by gas chromatography and mass spectrometry. For each condition, lipids corresponding to 5 × 107 cells were injected. Data are retention times and integrated areas (in %). Composition is expressed as a mass percentage. ND: not detected.

The sterol pattern was deeply modified when the cells were grown for 48 h in the presence of tomatidine. The cells exhibited an intense decrease in 24-alkylatedsterols and a marked increase in desalkyl sterols, including zymosterol and cholesta-7,24-dien-3β-ol (Table). DMSO (vehicle) had no effect on sterol composition (not shown). The accumulation of zymosterol is consistent with an inhibition of 24-SMT. This enzyme is responsible for the incorporation of methyl groups at position 24 of sterol intermediates, which is an essential step for the production of ergosterol and other 24-alkylated sterols in protozoan parasites (Urbina et al. 1996 , Nes 2000 , de Souza & Rodrigues 2009).

## DISCUSSION

The antifungal effect of azasteroids has been known for quite a long time, but the recognition of these agents as efficient chemotherapeutics against trypanosomatids was only recognised recently (Burbiel & Bracher 2003). Natural azasteroids are found in plants as part of the defence barrier against fungi and yeasts. Azasteroids are steroidal alkaloids that are hydroxylated in the three position, conjugated with glycosides and referred to as saponins. Tomatine is the most abundant saponin in the tomato plant and its antifungal properties are well documented (Sandrock & VanEtten 1998, Ito et al. 2007 ). However, the defensive properties of the aglycone tomatidine have been neglected because its concentration is nearly 100-fold lower than tomatine.

Some pathogenic fungi have acquired the ability to hydrolyse the glycoside portion of the glycoalkaloid, which produces compounds with no toxic characteristics. Removal of the terminal glucose of α-tomatine yields β-tomatine, which exhibits little or no toxicity against some fungi (Sandrock & VanEtten 1998). Removal of all four sugars yields the aglycone tomatidine, which maintains toxic properties against fungi, such as *Neurospora crassa *[50% effective dose (ED_50_) 51 µM], *Aspergillus nidulans *(ED_50_ 81 µM), *Cryphonectria parasitica *(ED_50_ 81 µM) and* Stemphylium solani *(ED_50_ 300 µM) (Sandrock & VanEtten 1998). This study showed that tomatidine exhibited toxicity against *P. serpens*. However, tomatidine levels in the tomato fruit are too low to be an effective barrier against infection by this trypanosomatid. Tomatine was more effective against *Phytomonas* and it must be the more important defensin because of its elevated concentrations in green tomatoes. The tomatine content of tomatoes decreases during maturation. Roddick (1976) attempted to define the distribution in plant tissues and the dynamics of the synthesis and degradation of tomatine during ripening in a series of studies. Tomatine was found in the supernatant of suspensions of pericarp tissue of green tomato fruit and the expressed sap from intact tissues. This glycoalkaloid is synthesised in microsomal organelles and it accumulates in vacuoles and/or the soluble phase of the cytoplasm. The tomatine content of sap was 0.4 mM (Roddick 1976, Eltayeb & Roddick 1984). Tomato glycoalkaloids content depends on cultivar, fruit ripening stage and agricultural practices (Friedman 2002).

The action of tomatine, like other glycoalkaloids, is likely an efficient permeabilisation of the cell membrane as a consequence of the formation of complexes with steroids, such as polyene antibiotics or the induction of apoptosis mediated by reactive oxygen species (Ito et al. 2007). The leaking of PK from cells in *P. serpens* suggests that the primary mechanism of cell death is cell membrane disruption. Fig. 2A shows that no cells were found in the culture media after treatment with tomatine (50 µM). All cells precipitated to the bottom of the bottle. However, only 10 µM was used for the cell permeabilisation experiments. Part of tomatine likely interacts with sterols in the serum, which explains this difference in drug sensitivity.

In contrast, tomatidine inhibited the growth of *P. serpens* and caused important morphological and sterol level alterations instead of membrane leakage. Nakamura et al. (1999) determined that ergosterol is the main sterol component in *Phytomonas*. We did not detect this sterol in our cells. One likely reason for this discrepancy is the poor resolution of the thin layer chromatography used in the previous study. The complexity of sterol composition in the living organism can only be resolved using sensitive instrumentation, such as GS-MS, which was used in the present study.

Some of the remarkable effects of tomatidine on *Phytomonas* were a decrease in the amount 24-alkylated sterols (∆^7^-ergosterol, ∆^7^-sitosterol, ∆^7^-avenasterol, 24 ethyllophenol and 24 ethylidenelophenol) and an increase in proportion of nonalkylated sterols (cholesta-8,24-dien-3β-ol and its isomer cholesta-7,24-dien-3β-ol) (Table). This pattern is consistent with an inhibition of 24-SMT, which is the enzyme responsible for the addition of these alkyl groups to carbon C-24. The inhibition of 24-SMT of *Leishmania amazonensis* was also suggested as the primary mechanism of tomatidine toxicity on this parasite (Medina et al. 2012).

Tomatidine inhibits ergosterol biosynthesis in *Saccharomyces cerevisiae* and prevents cell growth (Simons et al. 2006). The decrease in ergosterols in *Saccharomyces* occurs in parallel with an increase in zymosterol, which was interpreted as an inhibition of 24-SMT (Simons et al. 2006). Notably, other structural analogues of tomatidine, such as solanidine and solasodine, which are naturally present in potatoes and eggplant, respectively, are growth inhibitors of the yeast-like alga *Prototheca* *wi-* *ckerhamii* (Mangla & Nes 2000). These authors showed an in vivo inhibition of sterol biosynthesis in this alga and inhibition of 24-SMT in a cell-free preparation.

Membrane sterols in trypanosomatids are present as a complex mixture of 24-ethyl sterols and 24-methyl sterols, with cholesterol as a minor component. Our results show an increase in cholesterol levels in parasites treated with tomatidine (Table). These parasites may attempt to counterbalance the altered sterol biosynthesis with a higher cholesterol uptake from the medium. These results are consistent with a previous study (Haughan et al. 1995, Medina et al. 2012) in which an inhibition of sterol biosynthesis increased the amount of exogenous cholesterol uptake. Exogenously acquired cholesterol in trypanosomatids may also presumably be incorporated into the membranes, but cholesterol apparently cannot totally substitute the endogenously synthesised 24-alkylated sterols (Roberts et al. 2003).

*Phytomonas* have a high proportion of C_24_-ethylated sterols, such as ∆^7^-sitosterol. ∆^7^-avenasterol and ∆^7^-sitosterol are present in some plants, including American ginseng (*Panax quinquefolium *L.) (Beveridge et al. 2002). The presence of C_24_-ethyl sterols in trypanosomatids was described previously (de Souza & Rodrigues 2009) and, in some cases, such as *T. cruzi *epimastigotes, these sterols represented up to 30% of the total sterols (Beach et al. 1986). The biosynthesis of these compounds involves the participation of a putative SMT2, which, as a rule, adds a second methyl group at C_24_. SMT2 exhibits distinct inhibitor sensitivities that were described previously in *T. cruzi* (Urbina et al. 1995). However, more studies are needed to assess the sensitivity of SMTs to tomatidine. Sterols of in the C_28_-ergostane (24-methyl group) and C_29_-stigmastane (24-ethyl) types are essential for trypanosomatids. Therefore, the introduction of the alkyl group at C_24_ of the sterol side chain must confer a desirable property that is needed for some essential cellular function (Haughan et al. 1995, Roberts et al. 2003). In addition to their altered sterol compositions, cells treated with tomatidine showed significant ultrastructural changes and membrane disorganisation, including the appearance of large vacuoles in the cytoplasm that localised to the posterior region of the parasite in some cases. Our results suggest that the main target of tomatidine in *P. serpens* is the 24-methylation reaction, which causes a depletion of the C_29_ and C_28_-sterols and an accumulation of C_27_-sterols.

Tomatine does not appear to be toxic when consumed orally in moderate amounts (Friedman et al. 2000a, b). The absence of a 5,6-double bond in the B-ring of tomatidine results in a much less toxic molecule in both pregnant and non-pregnant mice compared to the structurally similar solasodine (which contains such a double bond). Friedman et al. (2003) confirmed related findings by Gaffield and Keeler (1993, 1996) on the influence of the structure of glycoalkaloids and aglycones on teratogenicities in hamsters.

In conclusion, our results indicate that tomatine disrupts the plasma membrane, which causes death of the parasite and tomatidine interferes with the growth and ultrastructure of *P. serpens*, apparently due to the inhibition of alkylated sterols synthesis. There is a clear necessity for novel drugs against trypanosomatids and apicomplexa parasites to help control the diseases caused by these pathogens. Our previous study reported that tomatidine was toxic to the promastigote form of *L. amazonensis *(Medina et al. 2012). Additional studies are needed to determine whether other trypanosomatids are also sensitive to this alkaloid and whether they have synergistic effects with other antiparasitical drugs to evaluate the potential of tomatidine as an adjuvant in the treatment of parasitic diseases.
